# Torsion in Uterus: An Obstetrical and Gynaecological Emergency

**DOI:** 10.7759/cureus.54839

**Published:** 2024-02-24

**Authors:** Sukanya Singh, Surekha Tayade, Drashti Patel

**Affiliations:** 1 Obstetrics and Gynaecology, Jawaharlal Nehru Medical College, Datta Meghe Institute of Higher Education and Research, Wardha, IND

**Keywords:** female reproductive tract, uterine torsion, emergency, uterus, torsion

## Abstract

Uterine torsion is defined as torsion of the uterus along its longitudinal axis greater than 45 degrees. It is observed in all age groups of the reproductive period, in all parity groups, and at all stages of pregnancy. Torsion from 60 degrees to 720 degrees has been described. It is not possible to clarify why it occurs, but numerous abnormalities have appeared with uterine torsion. It is a rare complication that can result in placental abruption and intrauterine foetal death. Pregnancy, giant fibroids, and ovarian cysts are among the most common causes. Vague clinical attributes make diagnosis challenging pre-operatively and can be missed on routine ultrasound. Being a rare life-threatening condition, it necessitates a high level of concern for diagnosis and prompt intervention to optimise results. This review will help the healthcare worker to understand the various presentation of uterine torsion and their management by appropriately and timely diagnosing it.

## Introduction and background

Uterine torsion (UT) is the twisting of the uterus in its normal axis. It occurs on the transition between the cervix and corpus uteri, with dextrorotation occurring in two-thirds of diagnosed cases [1]. Although the majority of UT occurs at the time of pregnancy it exaggerates the congenital and physiologic rotations and obliquities of the normal uterus. In uncomplicated pregnancies, it typically occurs in the last trimester and can have severe perinatal and maternal consequences [2]. There have been no maternal deaths reported before 20 weeks, mortality rates of 17% reported between 20 and 28 weeks, 10% at 29 to 34 weeks, and 9% at term gestation [3]. UT in a gravid uterus leads to a higher perinatal mortality rate of around 12% [4]. UT can cause a variety of nonspecific symptoms. Indeed, the documented instances either presented with pain in the abdomen, bleeding per vagina, intestinal symptoms, or were asymptomatic completely. It is also worth noting that UT can sometimes be detected through birth obstruction. The extreme difficulty in diagnosing UT preoperatively stems from the variable presentation, compounded with its rare occurrence. It is usually treated with a prompt laparotomy, and the diagnosis is made intraoperatively most of the time, especially if the patient is asymptomatic at the time of admission. It is an emergency at any stage of a woman’s life, because as of now no clinical findings or biomarkers specific to UT are known, and as preoperative diagnosis is difficult, it can delay surgical intervention and result in severe complications. Usually, the diagnosis of UT occurs during surgery [5,6].

## Review

Different presentations of torsion in non-gravid uterus and their management

Torsion of the uterus is even rare in nongravid uteri. It can result in ischaemic damage to the uterus which is irreversible, and worsens the clinical condition. This condition has only been reported in a few cases in non-pregnant women, the first by The Times in 1861, and then in other cases throughout the 20th century [7].

Dutra et al. reported a case of a young girl, not attained menarche with the presentation of abdominal pain and on examination found out to be large abdominal mass on the epigastric region with further revealed to be a cyst, thin-walled near the cavity of the peritoneum. Later on, the cyst was found to be a teratoma of the left ovary. The ovary as well as the corpus of the uterus were twisted 360 degrees anticlockwise. Due to no blood supply both the structures were found to be infarcted and were treated laparoscopically by left salpingo-oophorectomy (SO) and partial hysterectomy [8]. Two similar reported cases in which the presenting complaint was abdominal pain and it was accompanied by a cyst in the ovary and removal of the uterus was performed with bilateral SO in a woman of 35 years old, while in another case of a 73-year-old female with the same complaints with rotation of 360 degrees of both uteri and the ovaries was treated by laparotomy, similar to the above case [9,10].

Another case of utero-ovarian torsion in a three-year-old girl with complaints of abdominal pain, distension, vomiting with a tumour in the pelvis and haemorrhagic infarcted tissue treated by right SO and laparotomy [11]. Ramaswamy et al. reported a case with pain in the abdomen in a nine-year-old patient, which on ultrasound revealed a mass in the ovary but at the time of laparotomy, it was found to be rotated 180-degree uterus with ischemic ovaries and was then managed by subtotal removal of the uterus with right SO [12].

In the case of ovarian cyst where the UT was diagnosed before the operation and in CT, post-menopausal women who had a complaint of abdominal pain, post to which laparotomy revealed ruptured endometrium with infarction in the walls of the uterus. The cause of infarction, rupture and torsion was thought to be because of ovarian cyst in this case [13]. Another case of the right ovarian case in a 55-year-old female presented with the same complaints as above with 1800-degree rotations of the uterus and later the uterus was completely removed along with bilateral SO [14]. In one case of pre-menarcheal girl with a history of pelvic pain with no other abnormality, later upon investigation found to be cervical agenesis, hematometra with 180 degrees horizontal UT and sacral entrapment and managed by hysterectomy [15].

Mohapatra et al. reported a case of UT of 180 degrees with juvenile granulosa tumour of the ovary in a five-year-old girl with bleeding per vagina and abdominal mass on the right side with increased inhibin-B and breast development which was further managed by SO and pelvic lymphadenopathy to preserve fertility [16].

Grover et al. reported three cases of abnormal uterus and adnexa which was diagnosed as congenital anomaly which was not usual generally. Upon further investigation, UT was found with the presentation of abdominal pain, dysmenorrhea and amenorrhoea in 10-, 14- and 17-year-old girls with torsion of 270, 90 and 180 degrees respectively, and managed by subtotal uterine removal in 10 years and fertility preservation in other two cases [17].

UT can also present with urinary retention as a presenting feature as in a case of 37-year-old women with a known myotonic dystrophy. A palpable mass was found during the examination. Upon ultrasonography (USG), it was found to be leiomyoma and during laparotomy, UT of 60 degrees was found which was further managed by SO and total uterus removal [18].

In more than 50% of cases, right-sided rotation is seen. UT mainly occurs near the transition zone of the cervix uteri and corpus. It is typically counteracted by broad and round ligaments. It is unclear why it arises; however, it has been discovered that it is usually due to any abnormality in the uterus or its neighbouring organ or it can be pathological [19].

Causes of Uterine Torsion

The large body of literature concerning the ailment comes from individual case reports, so the precise aetiology of UT remains unknown and appears unrelated to parity, gestational and maternal age. Intriguingly, Jensen’s series identified the transverse lie as the most frequent abnormal foetal presentation (72%).

Even so, in premenarcheal as well as in young menstruating women, the reason for UT was discovered to be abnormalities of the organ of reproduction or due to tumours in the ovary. UT in elder/postmenopausal females occur mainly because of leiomyomas of the uterus or due to tumours in the ovary, with leiomyomas being the cause in a lot of instances.

Recent reviews have inferred that UT usually occurs within a normal pelvis and is not associated with any pelvic pathology. Nonetheless, this particular case falls into the 30% of cases where a discernible cause is not identified. In this situation, the mother and the foetus are exposed to considerable risk. The most frequently associated maternal complications include maternal hemodynamic shock and complete or partial placental abruption. Fetal complications include foetal hypoxia, foetal antepartum haemorrhage, and the risk of foetal death [20].

Diagnosis

Diagnosis is commonly taken intraoperatively, owing to the poor correlation between clinical symptoms and classic radiological evidence.

Clinical feature of UT in non-gravid females differs, with no identified trend in symptoms in any age group. Absence of peritoneal signs is often seen in over 60 years old according to one report. Concerning data of laboratory, no identified biomarkers are there to specifically indicate UT. A case has been reported in which lactate dehydrogenase and the levels of creatinine phosphokinase gradually increased following the onset of symptoms. Identically, there has been one reported case in which the patient developed coagulopathy. However, these findings were not common in UT as per certain reports [21,22].

Torsion of the nongravid uterus is a rare condition. Delays in diagnosing this condition can be fatal, so a high level of suspicion is required.

Complications

Irrespective of age, UT can spread aggressively and become threatening to life. There have been reports of cases complicated by renal failure (progressive), shock; and the need for blood transfusions. Five of the aforementioned premenarchal and younger menstruating females lost their fertility post-surgery. Because of the non-specific clinical symptoms, the majority of the females were evaluated for a couple of days to months before being surgically treated, with or without a preoperative diagnosis of UT [23]. 

Torsion in gravid uterus: presentation, diagnosis and management

The gravid uterus rotates during the last trimester of pregnancy, which is normal. However, a UT which is pathologic and beyond 45 degrees is very seldom observed. Around one-fifth of UT cases have no known cause, in the rest of the cases, predisposing factors comprise malpresentation, myomas, anomalies of the uterus, adhesions in the pelvis, and cysts in the ovary.

Acute torsion interferes with the circulation in the uterus. The primary clinical signs comprise obstructed labour, bleeding per vagina, shock, etc. The major condition that needs to be differentiated is abruptio placentae. In several cases, the clinical condition worsens gradually, leading to a diagnosis of "acute abdomen." The definitive diagnosis is only made during the laparotomy procedure [24].

According to some reports, the results of a CT scan and an MRI can help identify this condition. Many features of this scan, like infarction/ischemia of pelvic mass, gas in the cavity of the uterus, or changes in pelvic masses in terms of position, indicate UT. Uterine whirl sign cannot be seen on USG but can be seen on CT scans and MRIs, which is the most distinctive and frequent finding. Most of the revealed cases included a CT examination as well as an MRI. Consequently, because of the modest proportion of announced cases, it is harder to examine which one is valuable for identifying the sign. Notwithstanding, the utilization of different media may help distinguish other trademark discoveries like ischemia or dead tissue of the uterus. As a result, MRI and CT are usually recommended. Whirl sign of the cervix of the uterus is a reliable diagnostic tool for UT, even though it is a rare condition and lacks specific symptoms and laboratory data. Conversely, UT should be scrutinised as a differential diagnosis for women with severe abdominal discomfort, because early diagnosis and management may conserve fertility and avert life-threatening consequences [25-27].

Laparotomy and detorsion are commonly used treatments for UT. A Caesarean section may be essential [28]. Its therapy relies on when the UT occurs, although laparotomy is required in every case.

It is also determined by the symptom severity, ischemia indications intraoperatively and the torsion duration. Normal pregnancy can be achieved by rotating the uterus in a pregnant state and laparotomy [29]. Surgical treatment improves maternal prognosis but still leads to significant perinatal mortality. UT can also lead to dystocia. The appropriate answer to this problem is a Caesarean section. If anatomic repositioning fails, an intentional posterior hysterotomy may be necessary for foetal birth [30]. Surgery can also be beneficial if quickly administered. Fertility can be retained in some instances. If the uterus cannot be de-torn, doctors may perform a posterior low transverse hysterotomy for delivery [31].

In Matsumoto et al. case, the predominant symptom was severe abdominal discomfort, and the uterine corpus was turned 360 degrees. The ovary of the left side was likewise twisted 360 degrees, and it was treated by total abdominal hysterectomy (TAH) and bilateral salpingo-oophorectomy. while in Arumugham et al. UT was in a clockwise orientation of 180 degrees, with symptoms such as congestion. The patient's symptoms improved after their successful attempts to detort the uterus. Congestion was eased, and an incision performed in the anterior portion of the uterus resulted in the birth of a healthy infant [32].

In two cases of severe uterine torsion, emergency Caesarean section was done as an urgent treatment. In one case, UT was caused by a rupture of the amniotic membrane, whereas in another, it was symptomless [33,34].

Legarth and Hansen described another instance of a primigravida female, a 30-year-old with uterine didelphys, where the torsion closed the delivery canal and the patient was handled by Caesarean section [35].

Kim et al. presented a case of a primigravida 34-year-old woman who had an emergency Caesarean delivery for worsened preeclampsia symptoms at the 34th gestational week. A substantial pelvic adhesion in the uterine wall on the posterior side caused the uterus to be turned right to 90 degrees [36].

When to Consider a Caesarean Section

Torsion of the uterus after Caesarean section might lead to an accidental posterior hysterotomy. It's crucial to reconstruct normal uterine architecture before making an incision. This maintains the uterine vessels. Therefore, diligent attention should be given, particularly to the anatomical features, before surgical incision [37,38]. Mendling et al. described a 29-year-old lady in the 26th gestational week who presented with 180-degree torsion, and the foetus died the next day because of acute immaturity [39]. Cook et al. described the instance of a torsion of the uterus which was pathological with 270 degrees coupled with necrosis of the adnexa and uterus together with abruption of the placenta and subsequent shock and foetal death [40].

Signs of Ischemia and Perfusion in UT

Daw and Saleh reported a case of 540-degree twisted UT in an obese female, with pelvic mass. The ovaries and the tubes also were torsed. Every twisted structure was gangrenous as well as infarcted and managed by bilateral SO and hysterectomy [41]. Another case by Chua et al. described another instance of a nulliparous elder female suffering from fibroid in the uterus for a very long and presented with pain in the right quadrant and pelvic mass post to which on ultrasound necrosis in the fundus of the uterus with bilateral adnexa were noticed along with torsed fundus and the fibroid serving as the pivot point [42]. Lahood et al. reported a case of a female with a bicornuate uterus in mid-trimester with foetal growth limitation combined with UT, presented with discomfort in the abdomen. This may be a symptom of ischemia of the placenta closely related to a torsed uterus. The fallopian tube along with ovaries were necrosed and managed by removal of the uterus with right SO. Alpha-fetoprotein was found to be raised in this case [43].

## Conclusions

Though UT is an uncommon illness, given the potential for major morbidity, as well as serious consequences for fertility and is frequently identified during surgery, it should be considered one of the differential diagnoses in a woman with severe abdominal discomfort, myoma, or adnexal tumours. Techniques like CT/MRI scans and USG play an important role and should be interpreted with a high index of scepticism. Torsion is often treated with laparotomy and detorsion, but more severe instances may need hysterectomy or bilateral salpingo-oophorectomy. Patients with UT may have irrevocable necrosis, and ischemia, underscoring the importance of prompt radiological imaging. Timely intervention, mostly by immediate surgery, is critical since it can harm nearby organs such as the fallopian tube, spleen, and ovaries.
